# Predicting the Compressive Strength of Sustainable Portland Cement–Fly Ash Mortar Using Explainable Boosting Machine Learning Techniques

**DOI:** 10.3390/ma17194744

**Published:** 2024-09-27

**Authors:** Hongwei Wang, Yuanbo Ding, Yu Kong, Daoyuan Sun, Ying Shi, Xin Cai

**Affiliations:** 1 School of Resources and Safety Engineering, Central South University, Changsha 410083, China; hongwei.wang@csu.edu.cn (H.W.);; 2 China Construction Fifth Engineering Division Corp., Ltd., Changsha 410004, China

**Keywords:** sustainable cement–fly ash mortar, compressive strength, boosting machine learning, SHAP explanation

## Abstract

Unconfined compressive strength (UCS) is a critical property for assessing the engineering performances of sustainable materials, such as cement–fly ash mortar (CFAM), in the design of construction engineering projects. The experimental determination of UCS is time-consuming and expensive. Therefore, the present study aims to model the UCS of CFAM with boosting machine learning methods. First, an extensive database consisting of 395 experimental data points derived from the literature was developed. Then, three typical boosting machine learning models were employed to model the UCS based on the database, including gradient boosting regressor (GBR), light gradient boosting machine (LGBM), and Ada-Boost regressor (ABR). Additionally, the importance of different input parameters was quantitatively analyzed using the SHapley Additive exPlanations (SHAP) approach. Finally, the best boosting machine learning model’s prediction accuracy was compared to ten other commonly used machine learning models. The results indicate that the GBR model outperformed the LGBM and ABR models in predicting the UCS of the CFAM. The GBR model demonstrated significant accuracy, with no significant difference between the measured and predicted UCS values. The SHAP interpretations revealed that the curing time (T) was the most critical feature influencing the UCS values. At the same time, the chemical composition of the fly ash, particularly Al_2_O_3_, was more influential than the fly-ash dosage (FAD) or water-to-binder ratio (W/B) in determining the UCS values. Overall, this study demonstrates that SHAP boosting machine learning technology can be a useful tool for modeling and predicting UCS values of CFAM with good accuracy. It could also be helpful for CFAM design by saving time and costs on experimental tests.

## 1. Introduction

Cement is one of the most extensively used construction materials worldwide. In addition to being a building material, it can also be used as a binder in the stabilization/solidification of soil [1,2,3,4]. However, the highly energy-intensive and carbon-emitting production of cement has raised concerns [5]. Cement production accounts for around 5% of global CO_2_ emissions [6]. Some research focuses on cement production [7], while others focus on reducing cement use. It is suggested that environmentally friendly “supplementary cementing materials” (SCMs) be used to replace part of the cement used to produce mortar. Recently, SCMs such as silica fume, fly ash, powdered granulated blast furnace slag, and natural pozzolans have been employed [8,9,10].

Fly ash (FA) is a byproduct of coal-fired power stations burning pulverized coal to generate electricity [11,12]. FA has been used as a low-cost adsorbent [13]. A promising approach to improve the utilization of fly ash is to convert it into low-grade zeolites [14], which can be used in agriculture and many other fields [15]. In addition, because of its potential to increase the mechanics, impermeability, and durability of mortar and reduce carbon dioxide emissions, the use of FA in mortar has piqued interest in recent decades. The effect of FA on cement hydration and the pozzolanic reaction mechanism has been the subject of numerous investigations [16]. The unconfined compressive strength (UCS) of cement–fly ash mortar (CFAM) is contributed by the hydration reaction, packing effect, and pozzolanic reaction. The chemical composition of FA is widely acknowledged as one of the most critical parameters influencing CFAM performance [17]. Some research shows a high correlation between the CaO/SiO_2_ ratio and the UCS of CFAM, (CaO + MgO + R_2_O)/SiO_2_ ratio, and UCS of CFAM [11]. There are two main types of FA according to the ASTM standard C 618 [18]. If the SiO_2_ + Al_2_O_3_ + Fe_2_O_3_ content is more than 70 wt.%, this FA is classified as F fly ash. Meanwhile, FA is classified as C fly ash if it contains contents of between 50 and 70 wt.% of SiO_2_ + Al_2_O_3_ + Fe_2_O_3_.

One of the most essential performances of CFAM is its UCS [19,20]. The UCS of CFAM is usually obtained through an experiment in the laboratory. However, the experiment is time- and labor-consuming, because it involves lots of compressive tests for each type of FA and different curing times. Therefore, an approach that accurately predicts the UCS of CFAM is preferred. The UCS of CFAM is often predicted using traditional linear or nonlinear regression techniques on experimental data. Cyr et al. [21] proposed an empirical model to predict the UCS of CFAM. This model considered cement hydration, pozzolanic reaction, and the physical filling effect on the UCS, but the curing time was not considered. Qadir et al. [22] used a nonlinear relationship to quantify cement mortar’s compressive, split tensile, and flexural strengths as a function of the water-to-binder ratio (W/B), fly ash content, and curing time. However, with these empirical models, it is challenging to derive a precise regression equation. These equations’ generalization capabilities are also unsatisfactory, because they frequently only apply to a particular type of FA. More advanced techniques are required to replace traditional regression techniques in the UCS prediction of CFAM.

Machine learning has been used in various fields, like rock blasting and risk assessment [23,24,25,26]. In recent years, machine learning models have become the most popular technique for predicting compressive strength and other parameters in civil engineering [27,28,29]. These machine learning models include multiple linear regression, M5P tree model, support vector machine, decision trees, Random Forest, gene expression programming, and artificial neural networks [30]. Only a few studies predicted the UCS of CFAM using machine learning techniques. Mohammed et al. [31] used linear and nonlinear regression, M5P tree, and artificial neural network technical approaches to predict the UCS of CFAM, with the parameters of the fly ash incorporation ratio, water-to-binder ratio, and curing time. Moreover, as reported in [32,33], developing ensemble machine learning models is useful as it integrates the advantages of different models. The three main ensemble machine learning algorithm categories are bagging, boosting, and stacking [33].

Among them, boosting machine learning models have been widely used in the field of biomedicine [34,35], and they have higher prediction accuracies than other individual machine learning models [36]. Boosting is an ensemble machine learning technique that adjusts the sample weights of the next model’s learning data based on the previous model’s learning outcomes. It connects numerous weak learners to create strong learners. As a result, the results of the previous learning will affect the next learning, and the weights of the data will also increase with the learning time. However, the use of boosting machine learning models is not extensively reported in the literature related to the civil engineering field [37,38]. In particular, to the best of the authors’ knowledges, no studies focus on predicting the UCS of CFAM with boosting machine learning models. Moreover, these studies’ developed machine learning models are “black boxes” and not explainable. Thus, although these models have high accuracy, a user cannot understand their working principles, and this problem limits the application of machine learning models in civil engineering practice. Lundberg and Lee [22] recently developed a SHapley Additive exPlanations (SHAP) framework to interpret machine learning models. The SHAP approach could assess the contribution by features to the predictions quantitatively. It could explain how the features affect the machine learning models in both global and local views. Only a few studies have used SHAP to explain a developed machine learning model in civil engineering [39,40].

Therefore, in this study, we focus on developing three typical explainable boosting machine learning models—gradient boosting regressor (GBR), light gradient boosting machine (LGBM), and Ada-Boost regressor (ABR)—to predict the UCS of CFAM. Secondly, we use SHAP to explain the best model in this study, with global and local explanations. Lastly, we check the importance of input parameters, especially the chemical composition of FA, for the models. Ultimately, we compare the prediction accuracy of the best boosting machine learning model and the other commonly used machine learning models.

## 2. Machine Learning Techniques

The UCS of CFAM was determined using a variety of boosting machine learning techniques, including gradient boosting regressor (GBR), light gradient boosting machine (LGBM), and Ada-Boost regressor (ABR). In the end, the prediction performance of the best boosting machine learning model was compared with other commonly used machine learning models.

### 2.1. Gradient Boosting Regressor (GBR)

**Figure 1:**
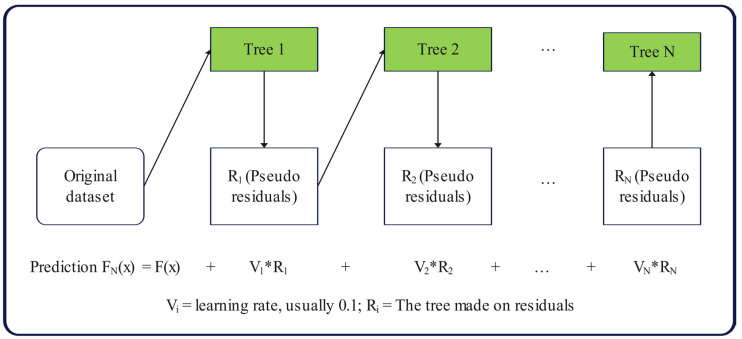
Diagram of the GBDT algorithm.

Friedman first proposed the GBR model as a robust and interpretable boosting model used for regression and classification [41]. The GBR is a boosting model that combines several weak learning models to create a robust predictive model. Decision tree models were used to build ensembles, and to correct the predictions produced by previous models, the number of trees was increased one at a time. Ending tree growth as soon as possible avoids the overfitting problem that decision tree learning faces. The GBR model is a powerful machine learning tool that can fit boosted decision trees by achieving a minimal loss gradient. It also offers improved performance and better stability. GBR provides excellent non-linear data prediction capabilities [42]. Figure 1 presents a diagram of the GBDT algorithm.

### 2.2. Light Gradient Boosting Machine (LGBM)

**Figure 2:**
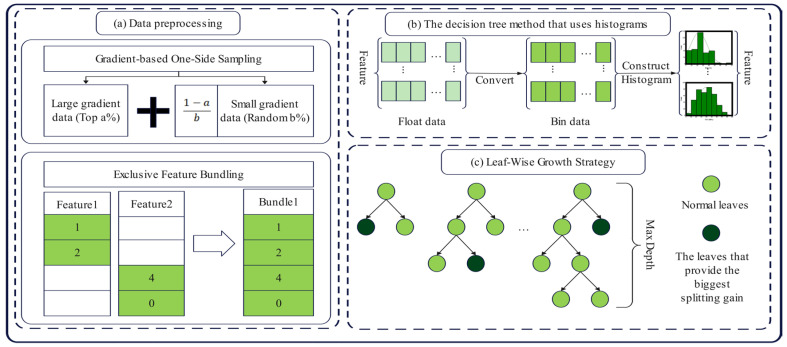
Diagram of the LGBM algorithm.

The LGBM was created by Microsoft in 2017 [43]. It is an ensemble boosting framework based on gradient boosting decision tree (GBDT). However, if the database contains a lot of data, both the prediction accuracy and the forecasting speed of the GBDT dramatically decline. In terms of memory consumption reduction and operating time acceleration while also maintaining high accuracy, LGBM improves the capabilities of GBDT [44]. According to reports [45], LGBM can accelerate GBDT’s training procedure up to 25 times while maintaining nearly the same accuracy. As a result, LGBM has become quite popular in machine learning because of its advantages of quick convergence speed and low memory utilization [46]. Figure 2 presents a diagram of the LGBM algorithm.

### 2.3. Ada-Boost Regressor (ABR)

**Figure 3:**
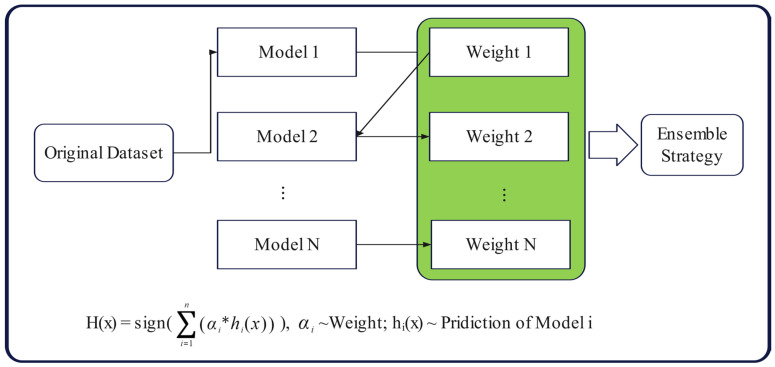
Diagram of the ABR algorithm.

Another boosting machine learning technique is the ABR, which was created by Yoav Freund and Robert Schapire [47]. It can randomly combine several weak learners from the dataset by weight to produce a boosted strong learner as the last result. The initial weight from the training set is used to train a base regressor, and the weight of the training sample is updated based on the learning error rate. For data points with high error rates to receive greater focus in the following base regression, the weights of the training sample points with high learning error rates in the prior base learner should increase. By changing the weight, the next base regression is trained, and so on, until the total number of base regressors reaches the predetermined number [32,48]. Figure 3 presents a diagram of the ABR algorithm.

### 2.4. Other Commonly Used Machine Learning Approaches

In addition, another ten commonly used machine learning approaches are used to compare against the best boosting machine learning model, including linear regression, Random Forest regressor, decision tree regressor, K Neighbors regressor, Bayesian ridge, ridge regression, extra trees regressor, least angle regression, Huber regressor, and lasso regression.

### 2.5. SHapley Additive exPlanations (SHAP)

Lundberg and Lee developed the SHapley Additive exPlanations (SHAP) in 2017 [49]. The SHAP is a Shapley game-theory-based method, which can be used to explain “black-box” machine learning models. The SHAP aims to assess the influence of each feature on the prediction results, and it can determine whether the influence of the feature is positive or negative. Accordingly, the SHAP can aid in explaining machine learning models from global and local views. In the meantime, the SHAP can conclude the contribution by each feature to each observation. The SHAP can aid in understanding the machine learning models’ underlying mechanisms and develop machine learning models that users can trust.

## 3. Model Development

**Figure 4:**
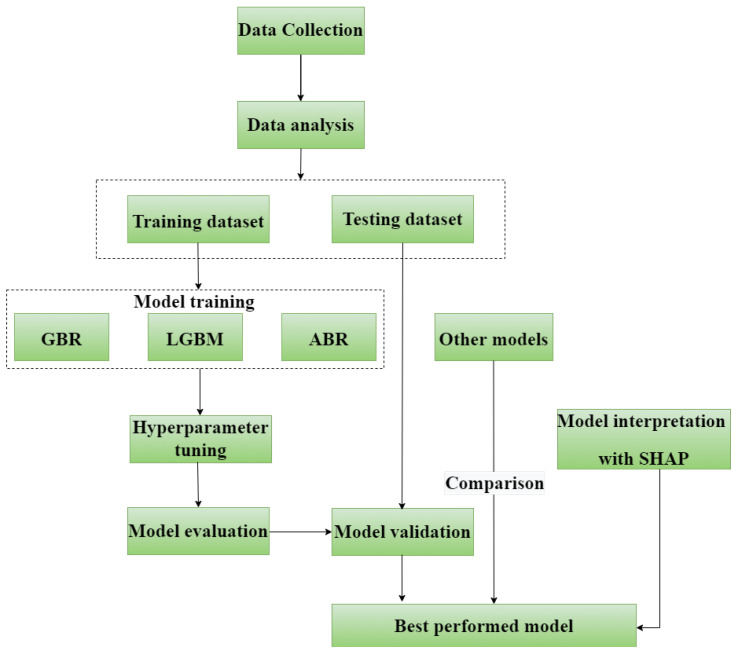
Workflow to develop models in this study.

In this study, we created all of the models using the Python platform (version 3.4). Figure 4 describes the entire proposed workflow for developing the models in this study, including five steps. The first step is data collection to build a database of UCS of CFAM. The second step is data analysis. The third step randomly divides all the data into two parts: 70% as a training dataset and 30% as a testing dataset. The fourth step is model training with the training dataset and hyperparameter tuning. The fifth step is model evaluation and validation with the testing dataset. Then, the SHAP method is used to interpret the best performed model. In the end, the best model is compared with other commonly used machine learning models.

### 3.1. Pretreatment of Data

It should be noted that, in this study, all data needed to be normalized with the Z-score normalization method so that the ranges were consistent. The database should be randomly divided into two groups, as follows: 70% of the data used for training the machine learning model and the remaining 30% of the data used for testing the machine learning model.

### 3.2. Cross-Validation Accuracy

**Figure 5:**
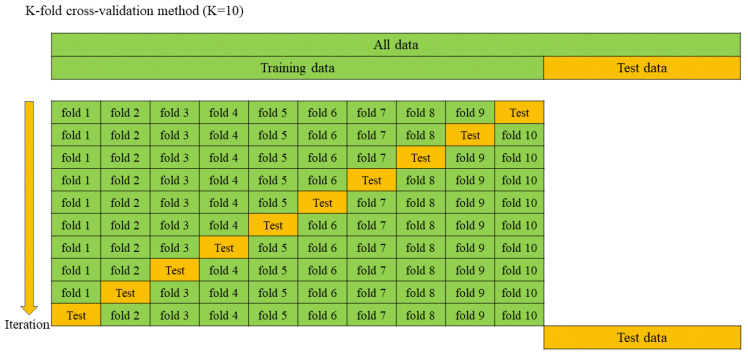
K-fold cross-validation method (K = 10).

The k-fold cross-validation approach enabled the model to be trained and validated multiple times, leading to a more accurate model with less overfitting [50]. Figure 5 shows a schematic description of the 10-fold cross-validation method. The training dataset was randomly divided into ten groups, of which nine were chosen for the model’s training and one was chosen for the model’s validation. Moreover, this process was repeated ten times, until each separate group was employed for validation.

### 3.3. Hyperparameter Tuning

Hyperparameter tuning is important in creating machine learning models, because the optimal hyperparameters can make the model more accurate. Hyperparameter values are usually chosen through a manual process of trial and error. However, manual tuning is frequently time-consuming and produces unsatisfactory results. Some automated approaches to identifying hyperparameters, such as grid search and random search hyperparameter optimization, have been developed to enhance the hyperparameter tuning process. This study used hyperparameter tuning for all machine learning models through the random grid search method.

### 3.4. Performance Evaluation of the Models

Five important parameters are frequently used to estimate and quantify a machine learning model’s performance, as follows: coefficient of determination ($R^{2}$), root mean square error (RMSE), mean absolute error (MAE), mean squared error (MSE), and mean absolute percent error (MAPE). The definitions of these parameters are shown in Equations (1)–(5).

(1) $$R^{2}=1-\frac{\sum_{k=1}^{n}{\left(y_{k}^{\prime }-y_{k}\right)}^{2}}{\sum_{k=1}^{n}{\left((y_{k}-\bar{y_{k}}\right)}^{2}}$$

(2) $$RMSE=\sqrt{\frac{\sum_{k=1}^{n}{\left(y_{k}^{\prime }-y_{k}\right)}^{2}}{n}}$$

(3) $$MSE=\frac{\sum_{k=1}^{n}{\left(y_{k}^{\prime }-y_{k}\right)}^{2}}{n}$$

(4) $$MAE=\frac{\sum_{k=1}^{n}\left|y_{k}^{\prime }-y_{k}\right|}{n}$$

(5) $$MAPE=\frac{\sum_{k=1}^{n}\left|{(y}_{k}^{\prime }-y_{k})/y_{k}\right|}{n}$$

where $y_{k}$ is the experimental value indexed with k; $y_{k}^{\prime }$ is the predicted value indexed with k; $\bar{y_{k}}$ is the mean of $y_{k}$; and n is the total number of data samples.

## 4. Database

### 4.1. Data Collection

The database in this study was established with a total of 395 observations from 20 different independent research studies [5,8,11,51,52,53,54,55,56,57,58,59,60,61,62,63,64,65,66,67]. The mortar specimens in the literature are mostly cubes, and the UCS of the cylinder mortars were transformed into those of cube specimens with an empirical formula. Table 1 presents the ranges of this database’s input and output parameters. The input parameters consisted of the fly-ash dosage (FAD), water-to-binder ratio (W/B), sand-to-binder ratio (S/B), curing time (T), and main chemical composition of fly ash (CaO, SiO_2_, Al_2_O_3_, and Fe_2_O_3_). The output parameter is the unconfined compressive strength (UCS) of the Portland cement–fly ash mortar. 

### 4.2. Statistical Analysis

Table 2 shows the descriptive statistics of the parameters in the database used for the model’s development and validation, including minimum, maximum, mean, median, sum, and standard deviation values.

**Figure 6:**
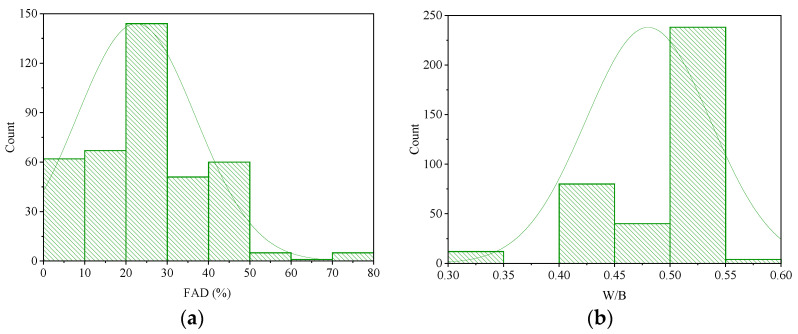

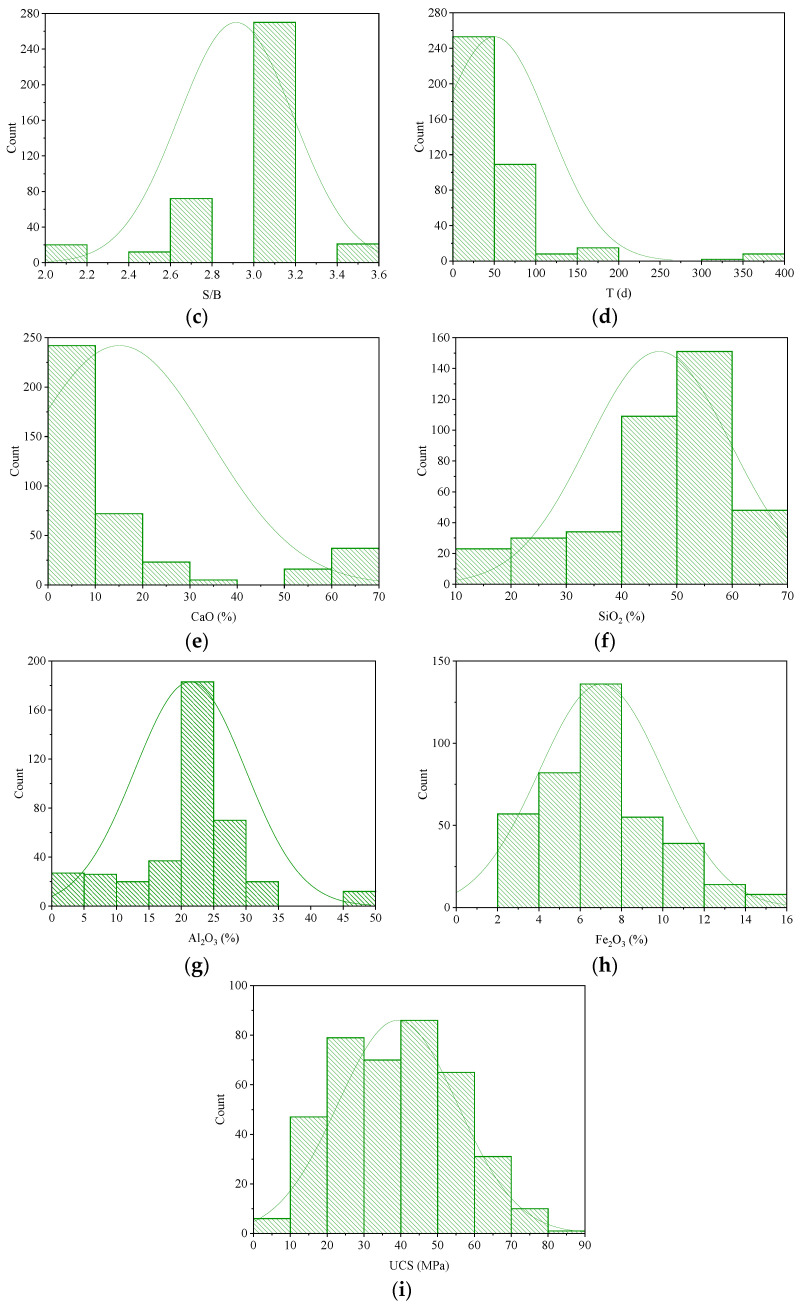
Histograms of the variables: (**a**) fly-ash dosage; (**b**) water–binder ratio; (**c**) sand–binder ratio; (**d**) curing time; (**e**) CaO content of the fly ash; (**f**) SiO_2_ content of the fly ash; (**g**) Al_2_O_3_ content of the fly ash; (**h**) Fe_2_O_3_ content of the fly ash; (**i**) unconfined compressive strength of Portland cement–fly ash mortar.

Figure 6 presents frequency histograms for each variable of the database. A few of the frequency histograms were discovered to be approximately normally distributed. The database included mortar samples with a wide range of FADs (0–80%) (Figure 6a), with the highest frequency of mortar samples containing 20–30% FA. The majority of the water-to-binder ratios (W/B) were in a range from 0.5 to 0.55 (Figure 6b). As shown in Figure 6c, the majority of the specimens had sand-to-binder ratios (S/B) that ranged from 3.0 to 3.2. In terms of the curing times (T), most values were found to be in the range of 0–50 d (Figure 6d), while the longest curing time was 365 d (Table 2). Regarding the chemical compositions of the FAs, more than 240 mortar samples were made with low-CaO-content (0–10%) FA(Figure 6e). SiO_2_ was the main chemical in the compositions of most of the used FAs (Figure 6f). The frequency distributions of the Al_2_O_3_ and Fe_2_O_3_ contents were more uniform, with the highest ranges being 20–25% (Figure 6g) and 6-8% (Figure 6h), respectively. The UCS values were normally distributed, varying from 7.5 to 71.5 MPa, with the highest frequency of 40–50 MPa (Figure 6i).

**Figure 7:**
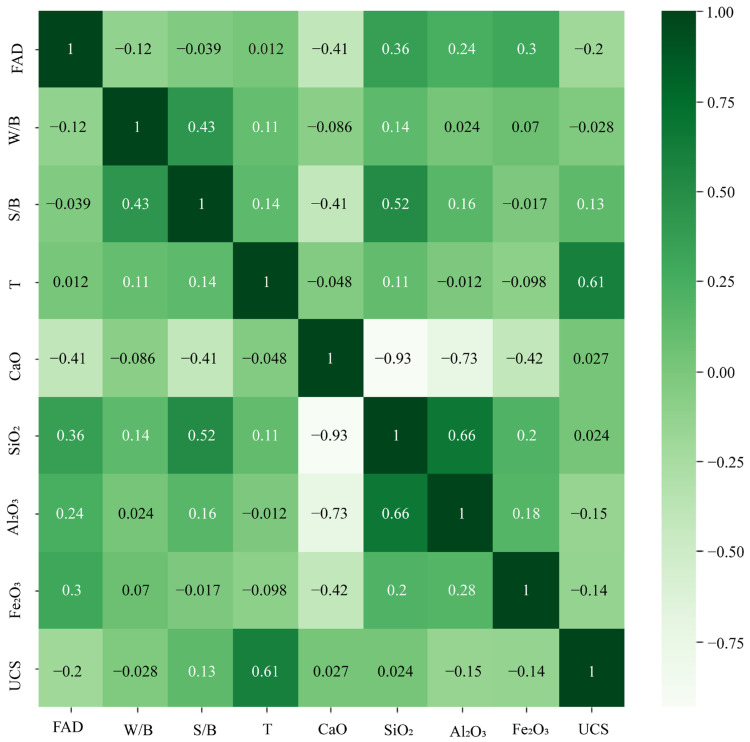
Heatmap of the correlations among the variables.

A correlation heat map is a method of perceiving information that displays correlated values along two dimensions using colors. This study presents the correlations between two variables in Figure 7, evaluating their linear dependence. It should be noted that some input variables had high correlations, as follows: CaO with SiO_2_ (R = −0.93) and CaO with Fe_2_O_3_ (R = −0.73). If two variables are correlated, only one variable should be used for modeling [68]. Therefore, in this study, it was decided that CaO should be eliminated. In this way, SiO_2_ and Al_2_O_3_ could be kept and used for modeling.

## 5. Results and Discussion

### 5.1. Hyperparameter Tuning and 10-Fold Cross-Validation

First, the random grid search procedure was used to find the optimal hyperparameters for each model. Table 3 lists the optimal hyperparameters and their associated values for each model. The models’ performances can be improved on the condition of these optimal hyperparameters.

**Figure 8:**
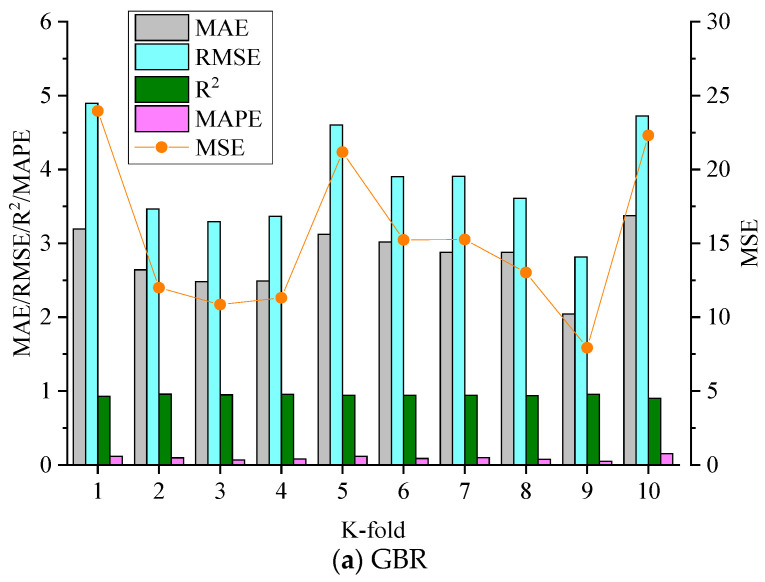

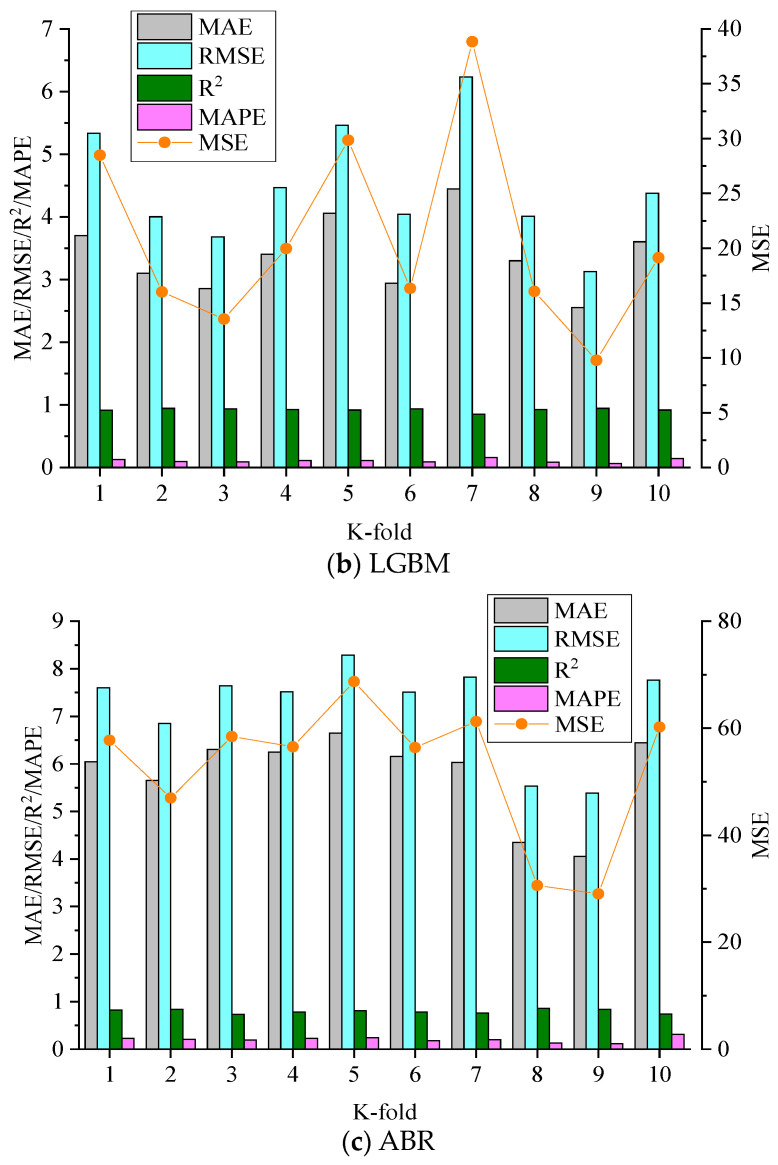
Results of the 10-fold cross-validation: (**a**) GBR model; (**b**) LGBM model; (**c**) ABR model.

To minimize the bias caused by the random sampling of the training dataset, a typical 10-fold cross-validation approach was used. According to reports [69,70], this approach can provide the models with generalization and reliability properties. Figure 8 illustrates the five error parameters of each fold in the 10-fold cross-validation, including MAE, $R^{2}$, RMSE, MAPE, and MSE. As can be observed, all three models’ 10-fold results exhibited fluctuations but still maintained high accuracy. Especially, Table 4 presents the average statistical results of the 10-fold cross-validation for the training dataset. It can be observed that the GBR model had fewer errors and higher *R*^2^ values than the LGBM and ABR models. For instance, the average *R*^2^ value of the GBR model over 10-folds was 0.941, and the maximum and minimum *R*^2^ values were 0.903 and 0.958. As for the LGBM model, the average *R*^2^ value over the 10-folds was 0.921, whereas the maximum and minimum values were 0.944 and 0.850, respectively. Similarly, the ABR model had average, maximum, and minimum *R*^2^ values of 0.796, 0.856, and 0.737, respectively.

### 5.2. Comparison of the Performances of the Different Models

**Figure 9:**
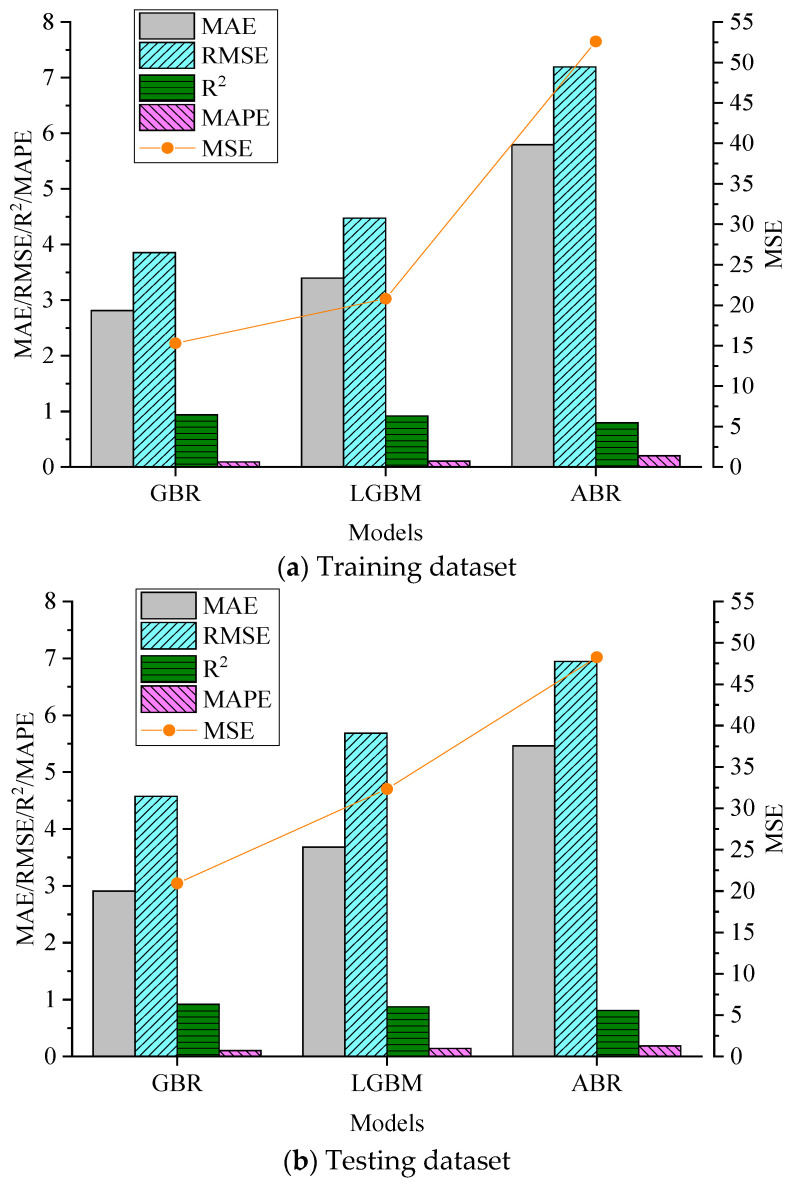
Performances of the optimized models on the (**a**) training dataset and (**b**) testing dataset.

The optimal hyperparameters shown in Table 3 were used to train all models on the training dataset, and the testing dataset was used to evaluate them. Figure 9 compares the models’ results on the training and testing datasets with the optimal hyperparameters. It is worth noting that the models’ performances on the training dataset was determined with a 10-fold cross-validation. The results demonstrate that the GBR model performed best on the training and testing datasets. It had the highest *R*^2^ value and the lowest *RMSE*, *MAE*, *MAPE*, and *MSE* values.

**Figure 10:**
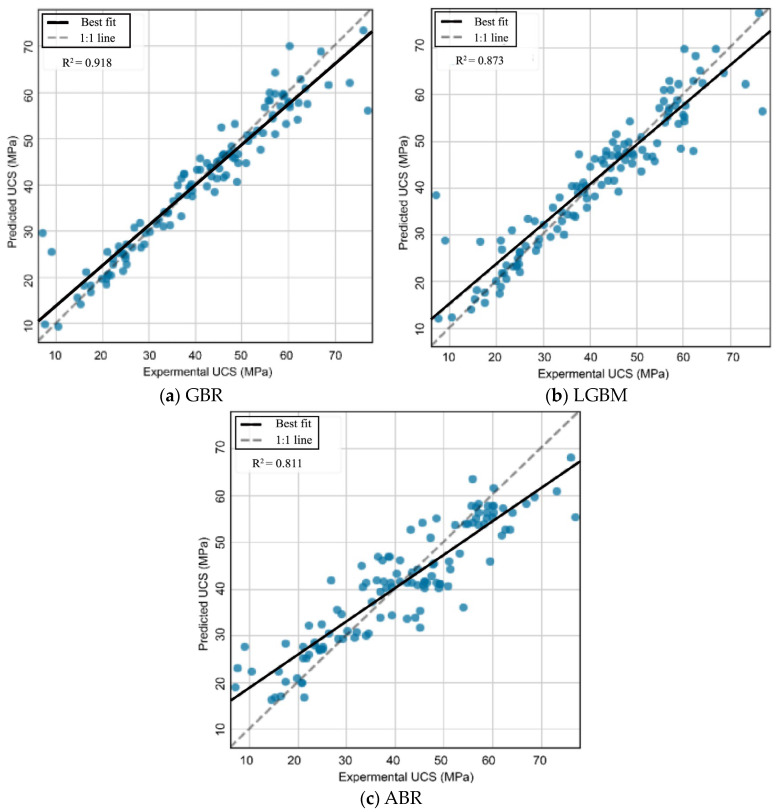
Relationship between the experimental and predicted compressive strengths: (**a**) GBR model; (**b**) LGBM model; (**c**) ABR model.

The best GBR, LGBM, and ABR models were created with the training dataset, and the validity of these models must be evaluated with the testing dataset. Figure 10 compares the experimental and predicted UCSs using the developed model for the testing dataset. It can be concluded that most of the predicted UCS values agreed with the experimental UCS values, for the best GBR and LGBM and ABR models. This indicates the great potential of these models for predicting the UCS of CFAM. The best GBR model had the highest prediction accuracy.

### 5.3. Residual Analysis

**Figure 11:**
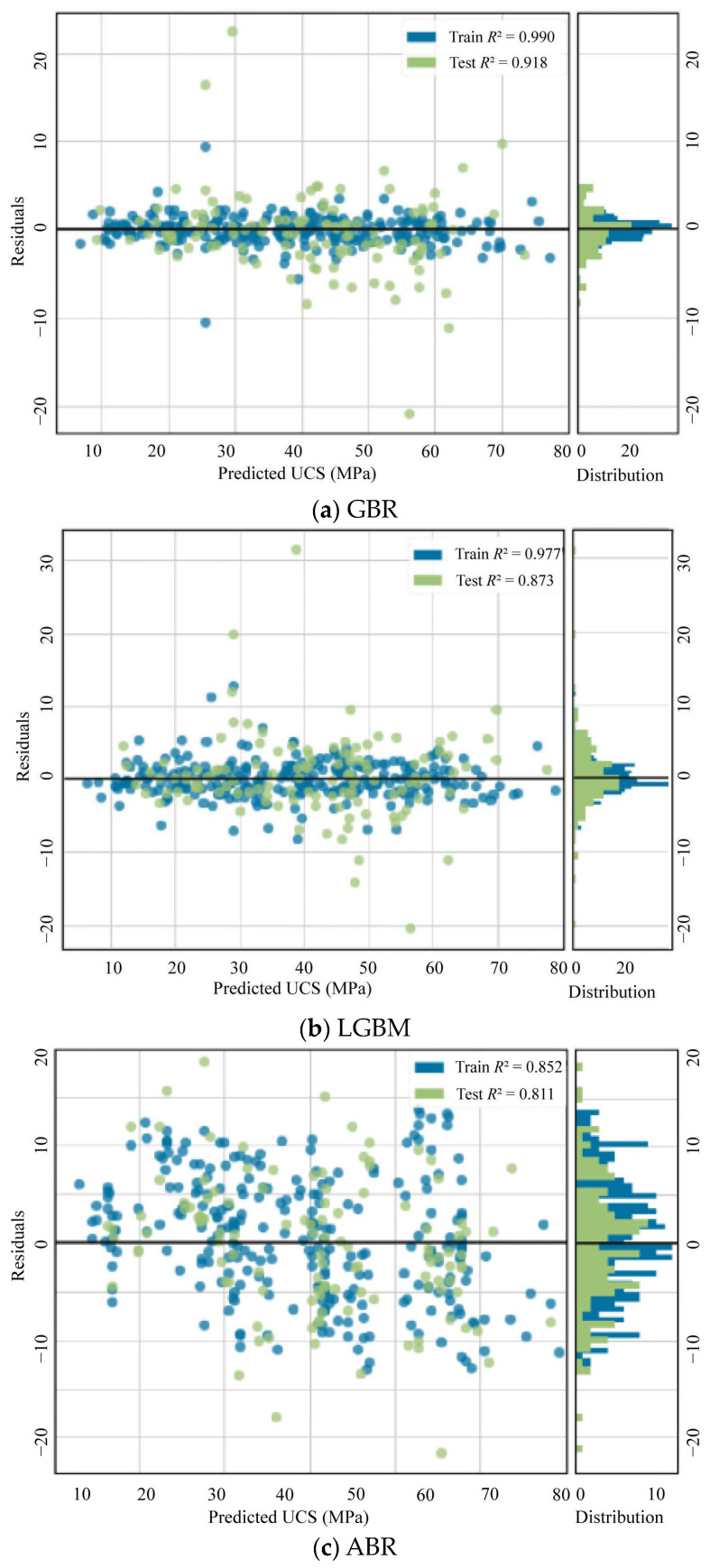
Residual analysis: (**a**) GBR model; (**b**) LGBM model; (**c**) ABR model.

In order to evaluate the adequacy of the best GBR, LGBM, and ABR models, the residuals for the best GBR, LGBM, and ABR models were statistically analyzed. Figure 11 shows the residuals versus predicted values for the best GBR, LGBM, and ABR models. The residuals seem to exist around the 0 line randomly for all of the best GBR, LGBM, and ABR models. This confirms that this study’s best GBR, LGBM, and ABR models have statistical significance. In addition, the best GBR model had the lowest residual values, indicating that the best GBR model is the best prediction model for the UCS of CFAM.

## 6. Interpretability and Feature Importance Analysis

**Figure 12:**
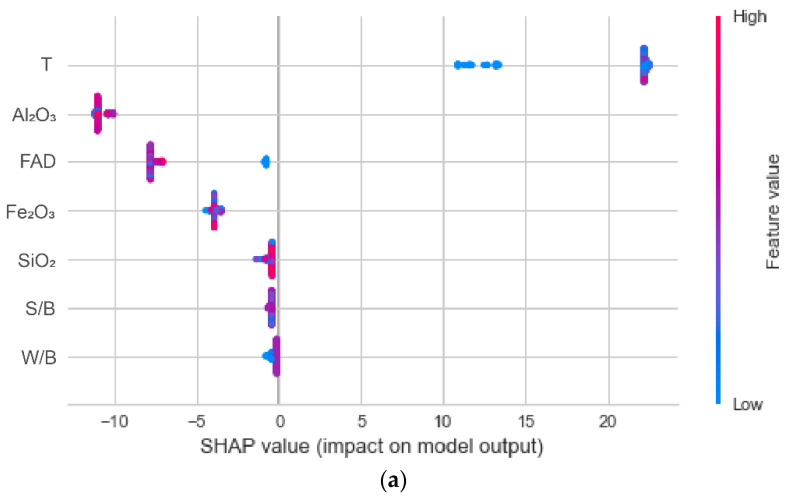

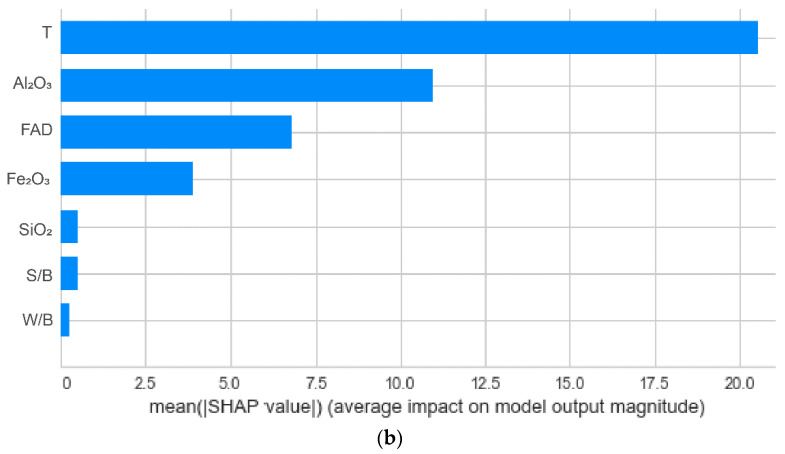
(**a**) SHAP global interpretation plot and (**b**) relative importance of each feature in the GBR model.

In this section, the SHAP method is used to interpret the best performing GBR model in order to investigate how the inputs influence the outputs of the developed GBR models. Figure 12a shows the SHAP summary plot. The SHAP value of each feature was represented by a dot in Figure 12a for a particular sample. The detailed SHAP values, which can be either positive or negative, are represented on the x-axis. The positive numbers indicate that the feature will raise the forecasts, while the negative ones suggest lowering them. Moreover, the feature values are coded by color, with blue representing lower feature values and red representing greater feature values. Figure 12b illustrates the feature of global importance factors. These values are the averages of the absolute Shapley values of each feature across the data. The more significant the feature, the higher the importance factor.

It can be found from Figure 12 that an increase in T results in an increase in the UCS, while the increases in Al_2_O_3_, FAD, and Fe_2_O_3_ cause the UCS to decrease. The rest of the features, like SiO_2_, S/B, and W/B, slightly impact the UCS. Significantly, the sand-to-binder ratio (S/B) is the least important variable in the GBR model. This indicates that changes in the S/B value play a very small role in predicting the UCS of CFAM. The curing time (T) was the most important variable in the GBR model. This indicates that changes in the T value play the most important role in predicting the UCS of CFAM with the GBR model. In addition, this analysis indicates that the chemical composition of FA, especially Al_2_O_3_, is a more important effect parameter than the fly-ash dosage (FAD) and water-to-binder ratio (W/B) in the prediction of the UCS of CFAM with the GBR model. As a highlight of this study compared with previous studies, this also proves that the chemical composition of FA is an important parameter influencing the UCS of CFAM. It is necessary to consider the chemical composition of FA as an input parameter in the prediction of the UCS of CFAM.

**Figure 13:**
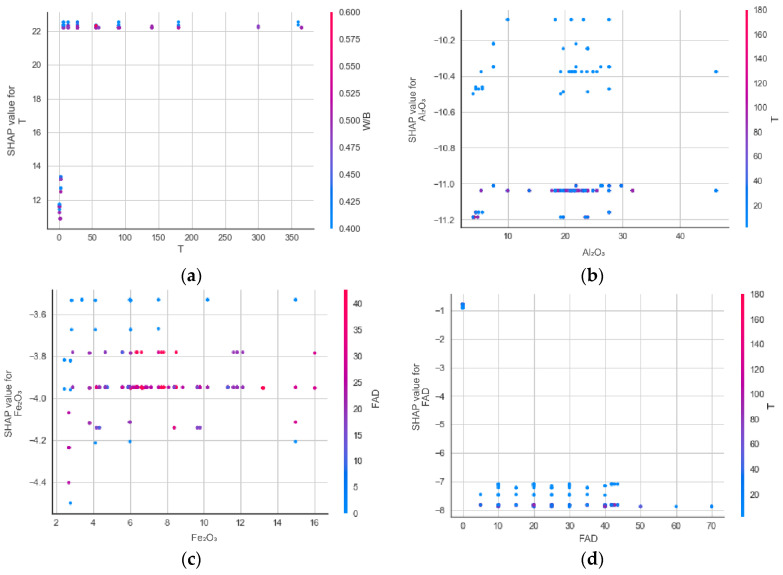

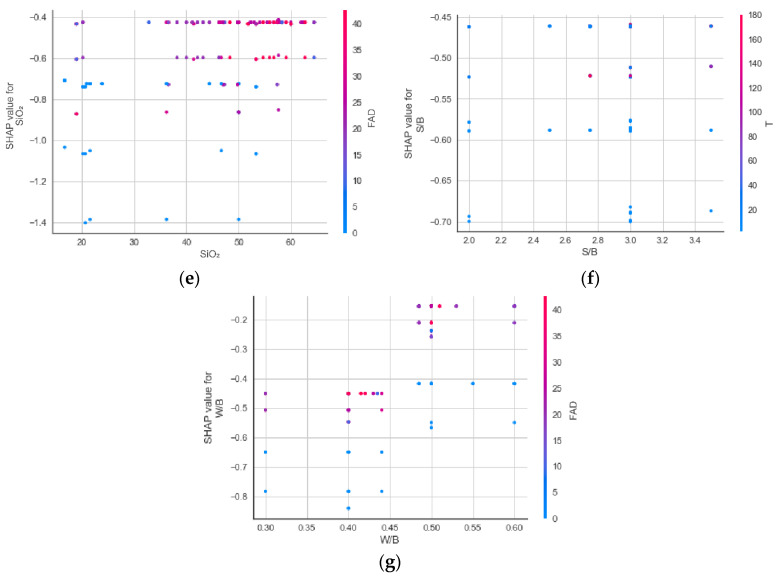
SHAP feature dependence plots: (**a**) T; (**b**) Al_2_O_3_; (**c**) FAD; (**d**) Fe_2_O_3_; (**e**) SiO_2_; (**f**) S/B; (**g**) W/B.

Figure 13 shows the SHAP feature dependency plot, which provides the SHAP value change for two features. The feature values and the corresponding SHAP values are shown on the x- and y-axes in Figure 13. The design variable most dependent on each characteristic, as shown in Figure 13, is represented by a colored bar on the plot’s right side. It should be noted that a high SHAP value indicates the model attempting to predict a high UCS value based on the corresponding feature values. In contrast, a low SHAP value indicates the model trying to output a low UCS value. For instance, as shown in Figure 10a, the dependency and interaction effects demonstrate that the features of 7 ≤ T ≤ 100 days and W/B ≤ 0.450 might result in high predicted UCS values.

**Figure 14:**
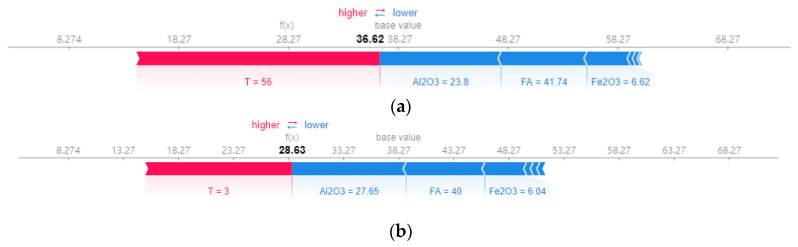
SHAP force plot of the selected two instances for local interpretation: (**a**) specimen No. 50; (**b**) specimen No. 187.

Figure 14 exhibits the GBR model’s local explanation with two selected specimens (No. 50 and No. 187). The base value represents the average experimental UCS value of 39.14 MPa. The bolded value of the (f(x)) represents the predicted UCS value. A red-marked feature (T) means an increase in the predicted UCS value over the base value. At the same time, blue-marked features (Al_2_O_3_, FAD, Fe_2_O_3_, SiO_2_, etc.) cause predicted UCS values to decrease to below the base value. For instance, for the specimen No. 50, as shown in Figure 11a, the predicted UCS value was 36.62 MPa. Following the order, the most important variables are T, Al_2_O_3_, FAD, and Fe_2_O_3_. Among them, a higher T will boost the predicted UCS value, while higher Al_2_O_3_, FAD, and Fe_2_O_3_ contents will drag the predicted UCS value down.

## 7. Comparison with Other Commonly Used Machine Learning Models

In addition, this study compared ten commonly used machine learning models with the best GBR model. Table 5 lists the performances of these different machine learning models using the training dataset. Similar to the GBR model, these models’ parameters are determined using similar approaches. As shown in Table 5, the best GBR model exhibited lower errors than the other ten commonly used machine learning models. This indicates that its predicted values were significantly closer to the experimental values. In comparison, it should be noted that the performance of the extra trees regressor (*R*^2^ = 0.877) and Random Forest regressor (*R*^2^ = 0.868) models can also be accepted and used to predict the UCS of CFAM. However, their errors were higher than those of the best GBR model. The other eight commonly used machine learning models did not provide satisfying accuracies. They had higher errors and lower *R*^2^ values. Therefore, the best GBR model in this study performed best in predicting the UCS of CFAM.

In engineering, the best GBR model can be used to select an appropriate type of FA and determine the approximate dosage range to reduce the time costs of preliminary tests and avoid UCSs below the limit due to inappropriate admixtures.

## 8. Conclusions

This study investigated and modeled the UCS of CFAM, with the following input parameters: chemical composition of fly ash, fly-ash dosage, water–binder ratio, sand–binder ratio, and curing time. The innovative aspect of this study is the development of boosting machine learning models (GBR, LGBM, and ABR models) to predict the UCS of CFAM. The best model was explained using the SHapley Additive exPlanations (SHAP) approach. Moreover, the best boosting machine learning model was compared with ten other commonly used machine learning models to better understand the performance of the boosting machine learning model. The following conclusions can be drawn:

(1) The GBR model performed better than the LGBM and ABR models and could be the best boosting machine learning model for predicting the UCS of CFAM.
(2) Compared to the other ten commonly used machine learning models, the GBR model exhibited significant accuracy in predicting the UCS of CFAM.
(3) The SHAP interpretations indicate that curing time (T) is the most important feature. The chemical composition of fly ash, especially Al_2_O_3_, is a more important effect parameter than fly-ash dosage (FAD) and water-to-binder ratio (W/B). The increase in T results in an increase in the UCS, while increases in Al_2_O_3_, FAD, and Fe_2_O_3_ cause the UCS to decrease.

In conclusion, this study shows the strong power of boosting machine learning models for predicting the UCS of CFAM. The better prediction performances of the boosting machine learning models indicate their importance in civil engineering. The best GBR model in this study could help engineers or researchers predict the UCS of CFAM under the conditions of different curing times, different types of fly ash, different fly ash contents, and different water-to-binder ratios.

## Figures and Tables

**Table 1:** Ranges of all data from the literature.

No.	FAD	W/B	S/B	T	CaO	SiO_2_	Al_2_O_3_	Fe_2_O_3_	UCS	Reference
	%			d	%	%	%	%	MPa	
1	0–30	0.50	3.00	3–28	9.37	46.70	19.21	7.55	15.52–53.27	[5]
2	0–35	0.50	3.00	1–140	6.30	50.00	23.90	6.00	12.30–62.30	[8]
3	25	0.50	3.00	28–91	2.54–6.17	52.20–62.60	17.70–23.00	8.85–6.15	41.10–69.50	[11]
4	42–44	0.50	3.00	3–365	3.10–6.49	48.30–62.70	21.20–25.50	6.34–8.5	21.10–71.50	[51]
5	5–25	0.50	3.00	7–180	2.00	58.30	31.70	5.90	10.40–33.00	[52]
6	40–70	0.40	2.75	7–28	1.61	51.80	26.40	13.20	7.50–36.00	[53]
7	10–30	0.50	2.50	3–28	18.10	46.25	46.25	5.60	9.00–41.10	[54]
8	10–50	0.42–0.44	3.00	7–90	0.98	60.02	29.77	6.68	8.31–53.93	[55]
9	0–30	0.50	3.00	1–28	20.17	36.19	19.67	14.96	6.88–49.75	[56]
10	0–40	0.51–0.55	2.75	7–180	13.00	44.40	23.50	10.20	29.00–77.00	[57]
11	0–40	0.30–0.40	3.00	3–90	2.86	53.33	27.65	6.04	21.36–80.38	[58]
12	20	0.49	2.75	3–90	13.20–63.80	20.20–43.20	5.40–21.50	2.90–12.10	15.00–44.50	[59]
13	0–40	0.50	2.75	7–90	14.40–65.40	20.90–41.10	4.80–21.60	3.40–11.30	33.00–63.50	[60]
14	0–30	0.44	3.00	3–28	6.10–62.87	20.21–41.40	4.94–26.20	2.85–16.00	15.91–46.25	[61]
15	0–40	0.40	2.00	1–28	51.29–61.87	18.95–20.65	5.60–7.53	3.82–4.13	11.20–44.90	[62]
16	0–10	0.49	2.75	7–300	39.69–61.00	23.84–32.80	4.20–13.77	3.40–4.78	29.42–66.92	[63]
17	0–20	0.60	3.50	3–180	1.68–66.40	16.70–64.45	3.97–24.83	2.46–4.67	24.37–43.85	[64]
18	0–25	0.40	3.00	1–360	3.87–62.83	21.56–57.60	4.44–21.90	2.70–2.78	7.00–74.95	[65]
19	10–30	0.50	3.00	2–90	8.11	47.26	27.63	4.35	12.70–57.00	[66]
20	15–35	0.50	3.00	2–90	2.99–25.72	36.56–57.39	10.00–23.20	4.20–9.65	10.60–61.10	[67]

**Table 2:** Descriptive statistics for the database.

	Mean	Median	Standard Deviation	Min	Max	Sum
FAD (%)	22.49	20.00	14.39	0.00	70.00	8883.00
W/B	0.48	0.50	0.06	0.30	0.60	189.68
S/B	2.92	3.00	0.28	2.00	3.50	1151.50
T (d)	50.07	28.00	65.97	1.00	365.00	19,776.00
CaO (%)	15.08	6.30	19.10	0.98	66.40	5956.44
SiO_2_ (%)	46.81	50.00	12.90	16.70	64.45	18,489.05
Al_2_O_3_ (%)	21.39	21.90	8.57	3.97	46.25	8449.17
Fe_2_O_3_ (%)	7.01	6.22	3.02	2.46	16.00	2770.45
UCS (MPa)	39.14	39.30	16.26	6.88	80.38	15,459.61

**Table 3:** Optimal hyperparameters of the used models.

Hyperparameter	Models		
	GBR	LGBM	ADA
n Estimator	170	120	220
Learning rate	0.05	0.3	0.3
Max depth	11	−1	-
Subsample	0.3	1.0	-

**Table 4:** Average statistical results of 10-fold cross-validation.

Models		*MAE*	*RMSE*	*R* ^2^	*MAPE*	*MSE*
GBR	Mean	2.813	3.857	0.941	0.094	15.303
	SD	0.380	0.652	0.016	0.028	5.144
LGBM	Mean	3.397	4.474	0.921	0.108	20.805
	SD	0.546	0.889	0.026	0.027	8.422
ABR	Mean	5.793	7.192	0.796	0.203	52.588
	SD	0.837	0.929	0.041	0.054	12.466

**Table 5:** Comparison with other various machine learning models.

No.	Model	*MAE*	*RMSE*	*R* ^2^	*MAPE*	*MSE*
1	Best GBR	3.423	4.594	0.917	0.113	21.720
2	Extra Trees Regressor	4.118	5.562	0.877	0.126	31.575
3	Random Forest Regressor	4.260	5.759	0.868	0.135	33.667
4	Decision Tree Regressor	5.668	7.802	0.754	0.172	63.521
5	K Neighbors Regressor	7.916	10.011	0.606	0.288	102.230
6	Bayesian Ridge	10.259	12.298	0.404	0.375	153.298
7	Ridge Regression	10.249	12.296	0.404	0.373	153.248
8	Linear Regression	10.248	12.297	0.403	0.373	153.279
9	Least Angle Regression	10.248	12.297	0.403	0.373	153.279
10	Huber Regressor	10.282	12.374	0.394	0.372	155.150
11	Lasso Regression	10.558	12.610	0.374	0.393	161.729

## Data Availability

The authors confirm that the data supporting the findings of this study are available within the article.
